# Sexual and reproductive health among men with genital schistosomiasis in southern Tanzania: A descriptive study

**DOI:** 10.1371/journal.pgph.0002533

**Published:** 2024-03-27

**Authors:** Twilumba Makene, Abdallah Zacharia, Stanley Haule, Gift Lukumay, Billy Ngasala

**Affiliations:** 1 Department of Parasitology and Medical Entomology, Muhimbili University of Health and Allied Sciences, Dar es Salaam, Tanzania; 2 Department of Pathology, Muhimbili University of Health and Allied Sciences, Dar es Salaam, Tanzania; 3 Department of Community Health Nursing, Muhimbili University of Health and Allied Sciences, Dar es Salaam, Tanzania; Georgetown University, UNITED STATES

## Abstract

Male genital schistosomiasis (MGS) is a significantly neglected condition, and its consequences often receive inadequate attention. The disease is suggested to cause schistosomiasis-induced sexual and reproductive health problems among males. The study was conducted to investigate the prevalence of MGS, sexual and reproductive health problems that could be caused by MGS among adult males in Mtama district. A community-based cross-sectional study using quantitative methods was carried out among males aged ≥ 18 years in selected households. Semen and urine samples were collected from each participant to establish the prevalence of MGS and urogenital schistosomiasis respectively. Semen quality was macroscopically and microscopically assessed. Urine samples were analyzed using filtration technique. A structured questionnaire interview was carried out to collect socio-demographic data, sexual and reproductive health information. Descriptive statistics were used to provide a summary of each variable. The prevalence (proportions) were presented in percentages and their respective 95% confidence intervals. A total of 223 adult males participated in this study. The prevalence of MGS and urogenital schistosomiasis were 5.8% (95% CI; 3.1%-9.0%) and 22.4% (95% CI; 16.6%-27.8%) respectively. The prevalence of *Schistosoma haematobium* eggs in semen was found high among young adults 12/129 (9.3%, 95% CI; 4.9%-15.7%), who never attended to school 6/35 (17.1%, 95% CI; 6.6%-33.6%), petty traders 4/26 (15.4%, 95% CI; 4.4%-34.9%), never impregnated woman 9/70 (12.9%, 95% CI: 6.6%-33.6%), experienced pain during ejaculation 4/17 (23.5%, 95% CI; 4.9%-15.7%), and with brownish semen 2/5 (40%, 95% CI; 4.9%-15.7%). According to the findings, MGS, like urogenital schistosomiasis, is prevalent in southern Tanzania. The disease is prevalent among males with some reproductive and sexual issues. This highlight the need for more research to investigate the association of MGS and male reproductive and sexual health for improved health services among males.

## 1 Introduction

Ending the neglect to attain sustainable development goals, the WHO published a road map for neglected tropical diseases (NTDs) 2021–2030. One of the proposed roadmap targets is the elimination of schistosomiasis as a public health problem and ultimately, the goal of eliminating transmission in all endemic countries by 2030 [1]. Schistosomiasis is a neglected parasitic disease caused by the trematode belonging to the genus *Schistosoma*. *Three Schistosoma* species are currently regarded as of a major public health importance. The species are *Schistosoma haematobium*; the main causative agent of urogenital schistosomiasis (UGS) in Africa and the Middle East, *Schistosoma mansoni*; the main causative agent of intestinal schistosomiasis in Africa, the Middle East, South America and the Caribbean and, *Schistosoma japonicum*; the main causative agent of intestinal schistosomiasis in East Asia (China, Philippines, and Indonesia) [2]. Globally, approximately 240 million people are infected (about 90% lived in sub-Saharan Africa) causing about 70 million disability-adjusted life years [3, 4]. Tanzania is ranked second to Nigeria for the countries with a high prevalence of schistosomiasis in Africa. More than 50% of the Tanzanian population lives in risk areas. Schistosomiasis is widely distributed in the country, whereby all regions are endemic with at least one of the two *Schistosoma* species; *Schistosoma mansoni* and *S*. *haematobium* [5].

*S*. *haematobium*, the main causative agent of UGS accounts for about 67% of all schistosomiasis cases in sub-Saharan Africa [6]. According to recent reviews, 32 out of 35 African countries with published data on schistosomiasis have reported UGS [2, 7]. The review that included only recent studies, the prevalence of UGS ranges from 0% to 67.4%, with the most reported prevalence rates for studies carried out between 2018 and 2022 falling between 10% and 20% [7]. UGS is caused by the lodging of *Schistosoma* eggs in the urinary tract (urinary schistosomiasis) or genital tract (genital schistosomiasis) [3]. Genital schistosomiasis occurring in female genitalia is called female genital schistosomiasis (FGS) while in males is called male genital schistosomiasis (MGS) [6]. It has been reported that the likelihood of UGS infection is higher in males than in females [8]. Estimations show that the number of men suffering from genital schistosomiasis is slightly higher than that of women. It is estimated that about 100 and 120 million people suffer from female genital schistosomiasis (FGS) and male genital schistosomiasis (MGS) respectively. Men are most often infected as they are more exposed to risk areas due to their occupation activities such as farming and fishing [9].

Historically, MGS was first reported by Professor Frank Madden in Egypt after he has described it in a reproductive organs and fluids of a 14 year Egyptian boy and an English soldier in 1911 [10]. Most of available evidences of MGS rely on post-mortem and case report (mainly on travelers) studies. In most endemic countries there are no or limited number of studies describing the epidemiology of MGS. The awareness and burden of MGS is often overlooked and underreported as its clinical presentations are usually similar to that of many sexually transmitted infections (STI) [11]. Few prospective studies conducted in endemic areas have reported significant prevalence of MGS among participants. The studies conducted in endemic areas of Malawi and Madagascar have significant proportion of participants with *S*. *haematobium* eggs in their semen samples [6, 12].

The pathological consequences of schistosomiasis in terms of intestinal, liver, and urinary presentations are well investigated and hence well-known, yet its chronic effect on host genitalia is rarely reported, probably overlooked and underestimated, and mostly overshadowed by the severe life-threatening and frequently fatal liver and renal affection [3, 6]. In recent days there is an increased interest in FGS, particularly after discovering an increased risk of HIV and HPV infection acquisition among women suffering from FGS [6]. Unlike FGS, MGS is severely neglected and their consequences often go unremarked at international and local levels. MGS is also a gender-specific manifestation of UGS which is poorly reported, understudied, and often misunderstood in most endemic areas [11].

Despite being severely neglected, MGS causes significant pathologies that may result in an increased risk of HIV infection and glandular tumors near the prostate (adenocarcinoma) among infected males [13]. In addition, once trapped from various blood vessels such as the superior mesenteric vein, the inferior mesenteric vein, spermatic veins, the deferential vein, the pampiniform plexus in the scrotum, pelvic veins that cross near the external inguinal ring and/or through direct spread from the epididymis to the testis [3, 14], *Schistosoma* eggs cause direct damage of testicular tissues by inflammation and granuloma formation [3]. Testicular schistosomiasis has been associated with infertility in endemic areas [15]. The eggs may also lead to intense granulomatous epididymitis and inhibition of spermatogenesis, causing male infertility [3, 14]. The symptoms of MGS include low backache, burning micturation, painful erection and ejaculation, weak erection or rapid (premature) ejaculation, azoospermia, oligospermia and haematospermia [11]. Majority of these symptoms were also associated to reproductive and sexual health complications [3, 14].

In spite of all these complications, the burden of MGS and the extent of morbidities that could be caused by it in most endemic areas including Tanzania remain under-researched or under investigated. Therefore, more research to better understand the impact of genital schistosomiasis in men is needed [16]. The main objective of this study was to determine the burden of MGS and the sexual and reproductive health problems that could be caused by MGS such as painful erection and ejaculation, weak erection or rapid (premature) ejaculation, azoospermia, oligospermia, and haematospermia among male of reproductive age in high endemic areas of Lindi region, Tanzania.

## 2 Materials and methods

### 2.1 Ethical statement

The study protocol was reviewed and approved by the Muhimbili University of Health and Allied Sciences Ethical Review Board (reference no. DA. 282/298/01.C/1048). Permission to conduct the study in the Mtama district was requested from the President’s Office—Regional Administration and Local Government, the Regional Administrative Secretary of the Lindi region, and the District Executive Director of Mtama district. In the beginning participants were given information about the study and details of the procedures that were performed, potential risks, and benefits involved. Their willingness to voluntarily participate in the study was sought, and written informed consent was obtained. Each participant received unique study identification number, and confidentiality was maintained. No participant names or any personal information were recorded. All participants were informed of their schistosomiasis testing results, and all *Schistosoma*-infected participants were offered free treatment (single 40mg/kg dose of Praziquantel).

### 2.2 Study area

**Fig 1:**
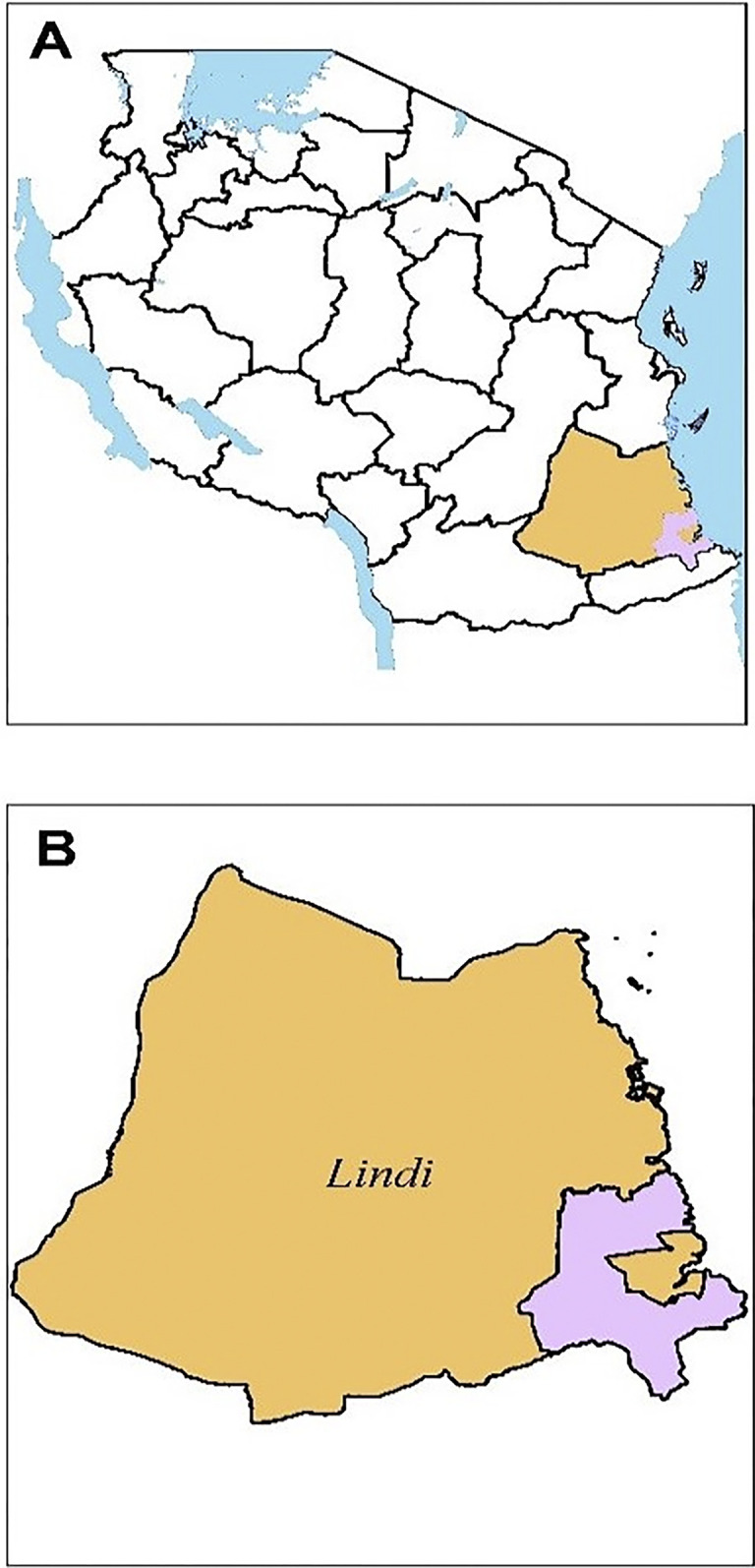
[A] Map of Tanzania showing the location of the Lindi region. [B] Map of the Lindi region showing the location of the Mtama district. Photo was adapted from Mushi *et al*., [18].

This study was conducted at Mtama DC. Mtama DC is one of the six district councils of the Lindi region. Lindi region is located in the southeastern zone of Tanzania (Fig 1). Mtama district council was formerly known as Lindi DC. The district council is boarded to the north by Kilwa DC, to the south by Mtwara region, to the west by Nachingwea DC, and to the east by the Indian Ocean and Lindi Municipal Council. Mtama district council has an area of 5975 km^2^ with an approximate population of 194,143 (102,496 females and 91,647 males). The ethnic groups are Mwera, Makua, Matumbi, and Ngido. The district experiences tropical climatic conditions characterized by an annual average rainfall of 910 mm and an average temperature of 26.3°C. The economic activities in the area are agriculture, livestock keeping, and fishing. Mtama DC has irrigation schemes which are used for agriculture and other domestic activities. The Mtama district is located in southern Tanzania, the area that has been reported among areas with a high prevalence of infertility [17]. The area has also been reported to have a high prevalence of UGS [18, 19]. The prevalence of UGS was significantly higher among boys than girls (57.2% vs 48.7%, p = 0.03) [18]. Three villages namely Mahumbika, Mtua longa and Nyengedi A were selected for this study.

### 2.3 Study design and population

A community-based quantitative cross-section study using quantitative methods of data collection was carried out in Mtama District Council (DC), Lindi region from May to June, 2022. Study participants were adult male community members living in highly schistosomiasis endemic wards identified and selected based on the findings of previous studies or surveys conducted in Mtama DC [18, 19].

### 2.4 Sample size estimation and sampling procedure

The sample size for this study was calculated by using the cross-sectional survey sample size calculation formula (n = z^2^ P (100-P)/ ε^2^, where n = sample size, z = standard deviation at 95% CL, P = expected prevalence and ε = margin of error) as described by Daniel [20]. The study used the prevalence of 10.4% recorded among the population of adult males in the neighboring country of Malawi [6], margin of error of 5%, standard deviation at 95% CI (1.96). The calculated sample size (n) was multiplied by the designing effect of 1.5 to obtain the actual sample size. The estimated sample size for this study was 239 adults after adjusting for a non-response rate of 10%.

The study participants were sampled through a three-stage cluster sampling technique. The first stage involved the purposive selection of one endemic district whereby the Mtama district council was selected among six district councils in the Lindi region. The second stage involved the selection of representative rural wards whereby out of the 11 rural wards, three UGS endemic wards (Nyengedi, Longa, and Kiwalala) were purposively selected. The third stage involved the selection of representative villages from the selected rural wards. From each rural ward, 1 village was selected by simple random sampling whereby Nyengedi A village was selected from the Nyengedi ward, Mtua Longa village was selected from the Longa ward, and Mahumbika village from the Kiwalala ward. The number of participants for each village was calculated based on the total number of adult males registered in each village. In each village, two community health workers (CHW) were provided with training on the study protocol. Then, the CHWs were asked to visit each household in the village that had males who met our predefined criteria to invite him to participate in the study. The invited participants were further screened if they met the study eligibility criteria before being provided with detailed information about the study and gave their written consent.

### 2.5 Eligibility criteria

The study included adult male community members in the selected villages aged ≥18 years, willing to provide written informed consent, stayed at the village for at least 12 months, and willing to give semen and urine samples. Participants who were unable to give sociodemographic information and received praziquantel treatment during the last three months were excluded from the study.

### 2.6 Data collection techniques and procedures

#### 2.6.1 Semen and urine samples collection and analysis

Participant were briefed about the study before obtaining written informed consent from each participant. For semen sample collection, study participants were asked to refrain from sexual activity including masturbation for three days before collection of semen samples. Each participant was given a choice to ejaculate either into a dry sterile plastic container or a sterile non-spermicidal condom [21]. Participants were required to submit semen samples within 60 minutes after collection [22]. For home sampling, a motorcycle was used to transport samples from distant participants’ houses. Participants were provided with pre-labeled urine containers and asked to put about 50 ml of urine between 10.00 am and 08.00 pm hours [23].

Semen samples were analysed as part of assessing the sexual and reproductive health of the participants and the determination of MGS infections. First, each semen sample was examined macroscopically. During this stage, the following information were collected; the time of collection and receipt of sample, the volume of the specimen, the colour (white, gray, and yellow) and the presence of blood in semen (haematospermia).

To determine semen viscosity, semen sample was aspirated into the Pasteur pipette. Then the semen was left to run out of the pipette back into the collection container. The length of the thread formed between the droplets was estimated in centimeter. To measure the semen pH, small droplet of semen was transferred on the semen strip using Pasteur pipette, then the color was allowed to develop. The color of the test strip was compared with its appropriate color scale depicted on the strip container.

To test for the sperm motility and presence of *Schistosoma* eggs, wet preparation of semen was prepared and examined under the microscope. Briefly, the semen sample was left to liquefy (within 15 to 30 minutes after collection, the semen liquefies by the action of fibrinolysis). After becoming fluid, one drop of semen sample was placed on a microscope slide and covered with a cover slip on it. The slide was examined under high power magnification (x40 objective). For sperm counting two or more fields were viewed. Both motile and non-motile spermatozoa were counted until a total of 200 spermatozoa were reached. The entire depth of a given field was focused so as to include spermatozoa that were settled to the bottom of the slide. Only spermatozoa that were moving forward were considered as motile. The percentage of motile spermatozoa were calculated and recorded. For detection of *Schistosoma* eggs, the whole smear was examined. To increase sensitivity for detection of *Schistosoma* eggs, two to three addition smears (depending on the volume of semen sample) were prepared and examined. As in spermatozoa examination, the entire depth of a given field was focused so as to include *Schistosoma* eggs that were settled to the bottom of the slide.

To count the number of spermatozoa, the liquefied semen sample was diluted with Sodium Bicarbonate formalin (1:20). The diluted sample was mixed and used to charge the Neubauer chamber. The Neubauer chamber was incubated to allow the mixture to settle. The spermatozoa were counted in four big squares. The spermatozoa were counted in the same manner as counting of white blood cells. The total number of spermatozoa was calculated by dividing the number of spermatozoa counted by the product of number of squares counted (four squares), depth of the chamber (20 μm) and the converted drop of semen in a ml of semen (1000 drops).

For morphological examination, a smear was prepared for each semen sample by adding a drop of egg albumin to the drop of semen on the slide and smearing with the second slide. The slide was air dried before being placed in a dark container containing fixative (an analytical-grade ethanol) until it arrived at the laboratory for staining (5–7 days). The slide was stained using a modified Papanicolaou stain method, as described by Björndahl L *et al*., [22]. Morphologically normal sperms were seen quite uniform in appearance. Any sperm with rounded, enlarged, small or bilobed head was regarded as abnormal. Also, sperm with tail such as enlarged, small, irregular in length or absent was considered as abnormal [22].

Urine filtration technique was used to examine the presence and quantify the number of *S*. *haematobium* eggs in urine. Briefly, the poly carbonate filter was placed in a filter holder, and then the urine sample was shaken and mixed before drawing 10 ml into a syringe. The filter holder was then attached to the end of a 10 ml syringe, and plunger of the syringe was pulled down to push all the urine through the filter to a bucket containing disinfectant. The syringe was detached from the filter unit, then unscrewed and with the use of tweezers the filter was removed and placed it inverted, onto the glass microscope slide. One drop of Lugol’s iodine was added in order to make eggs easily visible under the microscope. Slide was examined under a microscope using x40 objective. The eggs of *S*. *haematobium* was identified based on their characteristic large size and shape with a terminal spine. Infection loads was recorded as the number of eggs per 10 ml of urine. Intensity was established for all positive samples [23].

#### 2.6.2 Individual questionnaires

A structured questionnaire developed in English and then translated to Kiswahili was administered to all participants included in this study by a well-trained interviewer. The pre-tested structured questionnaires were administered to study participants to collect data on socio-demographic characteristics, sexual and reproductive health information, and schistosomiasis past history. Then, the filled questionnaire forms were back-translated to English for data entry and analysis.

### 2.7 Data analysis

We entered data into IBM SPSS version 20 for analysis. Continuous variables were summarized by mean and 95% confidence interval and categorical variables were summarized by frequency and percentage. The prevalence of MGS was calculated by taking the number of males with MGS per total number of participants and reported as a proportion (percentage). The prevalence of MGS was further summarized based on variables such as sociodemographic characteristics of participants and sexual and reproductive health variables. The prevalence (proportions) were presented in percentages and their respective 95% confidence intervals.

## 3 Results

### 3.1 Sociodemographic characteristics of study participants

Out of 239 invited adult males, 223 (93.3%) met the criteria and agreed to participate part in this study. The mean age was 34.2 years (95% CI: 32.4 years -36.0 years), with a range of 18 to 70 years. Table 1 describes each sociodemographic characteristic that was assessed. Many participants (57.8%) were young adults (≤ 34 years). Most of them (66.4%) completed a primary level of formal education, with only 15.7% having never attended any formal education. About half (46.2%) of participants were in a relationship or cohabiting, followed by married (40.8%) and those who were not in the relationship (widow, divorced, or single) accounted for 13% of all participants. The majority of participants were peasants (72.6%), who had lived in their respective villages for more than 10 years (92.4%). Many participants (43.5%) came from Mtua Longa village, followed by Nyengedi A village (29.6%) and Mahumbika village (26.9%).

**Table 1:** Demographic characteristics of the study participants (N = 223), in Mtama district, Lindi from May to June 2022.

Variable	Category	n	% (95% CI)
Age group	≤ 34 years	129	57.8 (51.6–64.6)
	35–49 years	56	25.1 (19.3–30.5)
	≥ 50 years	38	17.0 (12.1–22.0)
Education	Never	35	15.7 (11.2–20.2)
	Primary	148	66.4 (59.7–72.6)
	Secondary and above	40	17.9 (12.6–22.9)
Marital status	Married	91	40.8 (34.5–48.0)
	Divorced/Widower/Single	29	13.0 (9.0–17.9)
	In relationship/cohabiting	103	46.2 (39.5–52.9)
Occupation	Peasants	162	72.6 (65.9–78.5)
	Petty business	26	11.7 (7.2–16.1)
	Others	35	15.7 (11.2–20.6)
Village of residence	Mahumbika	60	26.9 (21.1–32.7)
	Mtua Longa	97	43.5 (36.8–50.2)
	Nyengedi	66	29.6 (24.2–35.4)
Residency	≤ 5 years	14	6.3 (3.6–9.4)
	6–10 years	3	1.3 (0.0–3.1)
	˃ 10 years	206	92.4 (88.8–100.0)

n: number of participants in each category

### 3.2 Prevalence of male genital schistosomiasis and urogenital schistosomiasis

Thirteen (5.8%, 95% CI; 3.1%-9.0%) of 223 participants had ***S. haematobium*** eggs in their semen. The prevalence of ***S. haematobium*** eggs in semen was significantly higher among young adults (9.3%, 95% CI; 4.9%-15.7%), those who had never attended school (17.1%, 95% CI; 6.6%-33.6%), and petty businesspersons (15.4%, 95% CI; 4.4%-34.9%). The prevalence was also high among participants who were in the relationship or cohabiting (8.7%, 95% CI; 4.1% - 15.9%), came from Nyengedi A village (7.6%, 95% CI; 2.5%-16.8%), and had lived in the village for more than 10 years (6.3%, 95% CI; 3.4%-10.6%) (Table 2).

**Table 2:** Prevalence of MGS by demographic characteristics of study participants (N = 223), in Mtama district, Lindi from May to June 2022.

Variable	Category	n (%)	Number of positive	% of positive (95% CI)
Age group	≤ 34 years	129 (57.8)	12	9.3 (4.9–15.7)
	35–49 years	56 (25.1)	1	1.8 (0.05–9.5)
	≥ 50 years	38 (17.0)	0	0.0 (0–9.0)
Education	Never	35 (15.7)	6	17.1 (6.6–33.6)
	Primary	148 (66.4)	6	4.1 (1.5–8.6)
	Secondary and above	40 (17.9)	1	2.5 (0.06–12.9)
Marital status	Married	91 (40.8)	2	2.2 (0.3–7.6)
	Divorced/Widower/Single	29 (13.0)	2	6.9 (0.8–21.4)
	In relationship/cohabiting	103 (46.2)	9	8.7 (4.1–15.9)
Occupation	Peasants	162 (72.6)	6	3.7 (1.4–7.9)
	Petty business	26 (11.7)	4	15.4 (4.4–34.9)
	Others	35 (15.7)	3	8.6 (1.8–23.1)
Village of residence	Mahumbika	60 (26.9)	1	1.7 (0.04–8.9)
	Mtua Longa	97 (43.5)	7	7.2 (3.0–14.3)
	Nyengedi A	66 (29.6)	5	7.6 (2.5–16.8)
Residency	≤ 5 years	14 (6.3)	0	0.0 (0.0–23.2)
	6–10 years	3 (1.3)	0	0.0 (0.0–70.7)
	˃ 10 years	206 (92.4)	13	6.3 (3.4–10.6)

n: number of participants in each category

The prevalence of UGS was 22.4% (95% CI; 16.6%-27.8%). Out of the 13 participants with ***S. haematobium*** eggs in their semen, 5 (38.5%, 95% CI; 11.1%-66.7%) had ***S. haematobium*** eggs detected in their urine samples (Table 3).

**Table 3:** Cross tabulation showing number of infected participants with male genital schistosomiasis and urogenital schistosomiasis in Mtama district, Lindi from May to June 2022.

		Male genital schistosomiasis		Total
		Positive	Negative	
**Urogenital schistosomiasis**	Positive	5	45	50
	Negative	8	165	173
**Total**		13	210	223

### 3.3 Reproductive and sexual health history and male genital schistosomiasis

The majority of participants (87.0%, 95% CI; 82.5%–91.0%) had female partners, 69.1% (95% CI; 62.8%–74.4%) had single partners, and 85.1% (95% CI; 79.6%–90.3%) had been with their partners for over one year. About one-third of the participants reported having never caused pregnancy (31.4%, 95% CI: 25.6%-37.7%). The majority of respondents (78%, 95% CI; 62.8%-74.9%) said they needed more children. MGS was significantly higher among participants who had never been responsible for impregnating woman (12.9%, 95% CI: 6.1% - 23.0%) and who did not have children (10.7%, 95% CI: 5.0% - 19.4%) (Table 4).

**Table 4:** Reproductive health history of the study participants (N = 223) and prevalence of MGS by reproductive health characteristic in Mtama district, Lindi from May to June 2022.

Variable	Category	n	% (95% CI)	Number of infected	% of infected (95% CI)
Had female partner(s)	Yes	194	87.0 (82.5–91.0)	11	5.7 (2.9–9.9)
	No	29	13.0 (9.0–17.5)	2	6.9 (0.9–22.8)
Number of partners	None	29	13.0 (9.0–17.9	2	6.9 (0.9–22.8)
	One	154	69.1 (62.8–74.4)	10	6.5 (3.2–11.6)
	More than one	40	17.9 (13.5–22.9)	1	2.5 (0.06–13.2)
Time with current partner	Less than 1 year	29	14.9 (9.7–20.4)	2	6.9 (0.9–22.8)
	1 year and above	165	85.1 (79.6–90.3)	9	5.5 (2.5–10.1)
Ever responsible for impregnating a woman	Yes	153	68.6 (62.3–74.4)	4	2.6 (0.7–6.6)
	No	70	31.4 (25.6–37.7)	9	12.9 (6.1–23.0)
Last time to impregnate a woman	Less than 1 year	14	9.2 (4.7–14.2)	0	0.0 (0.0–23.2)
	More than 1 year	139	90.8 (85.8–95.3)	4	2.9 (0.8–7.2)
Do you want more children?	Yes	174	78.0 (72.2–83.4)	12	6.9 (3.6–11.7)
	No	49	22.0 (16.6–27.8)	1	2.0 (0.05–10.9)
Do you use contraceptive?	Yes	6	3.1 (1.0–5.9)	0	0.0 (0.0–45.9)
	No	188	96.9 (91.4–99.0)	11	5.6 (2.9–10.2)
Did your partner use contraceptives?	Yes	91	46.9 (39.5–53.6)	5	5.5 (1.2–10.9)
	No	30	15.5 (11.1–21.9)	3	10.0 (5.3–32.8)
	I don’t know	73	37.6 (30.0–43.8)	3	4.2 (0.4–9.7)

n: number of participants in each category.

Out of 223 respondents, 212 (95.1%, 95% CI; 91.9%-97.8%) practiced sex, with 165 (77.8%, 95% CI; 71.5%-83.5%) reported to practice sex more than once per week and 202 (95.3%, 95% CI; 92.3%-98.1%) declared to enjoy it (Table 5). Among the 212 participants who reported having sex, 40 (18.9%, 95% CI; 13.5%-24.0%) said they couldn’t get an erection every time they wanted one. Sixteen (7.5%, 95% CI; 4.2%-11.2%) had erections that were insufficiently firm to place the penis in their partners’ bodies. A total of 77 participants (36.3%, 95% CL: 29.9%-42.7%) were unable to control their orgasm. Ten participants (4.7%, 95% CI; 2.3%-7.9%) reported pain during sexual intercourse, 5 (2.4%, 95% CI; 0.5%-4.7%) reported pain during an erection, and 17 (8.0%, 95% CI; 4.7%-12.0%) reported pain during ejaculation. The prevalence of MGS was significantly higher in participants who reported pain during ejaculation (23.5%, 95% CI: 6.8% - 49.9%).

**Table 5:** Sexual activities of participants (N = 223) and prevalence of MGS by sexual activity in Mtama district, Lindi from May to June 2022.

Variable	Category	n	% (95% CI)	Number of infected	% of infected (95% CI)
Do you practice sex?	Yes	212	95.1 (91.9–97.8)	13	6.1 (3.3–10.3)
	No	11	4.9 (2.2–8.1)	0	0.0 (00.0–28.5)
If yes, how often do you have sexual intercourse? (Per week)	< 1	12	5.7 (2.8–8.8)	1	8.3 (0.2–38.5)
	Once	35	16.5 (11.7–22.1)	2	5.7 (0.7–19.2)
	˃ 1	165	77.8 (71.5–83.5)	10	6.1 (2.9–10.9)
Do you have happy sex?	Yes	202	95.3 (92.3–98.1)	13	6.4 (3.5–10.8)
	No	10	4.7 (1.9–7.0)	0	0.0 (0.0–30.9)
Are you able to achieve an erection each time you would like to have one?	Yes	172	81.1 (76.0–86.5)	12	7.0 (3.7–11.9)
	No	40	18.9 (13.5–24.0)	1	2.5 (0.06–13.2)
Is your erection always firm to put penis in your partner’s body?	Yes	196	92.5 (88.8–95.8)	13	6.6 (3.6–11.1)
	No	16	7.5 (4.2–11.2)	0	0.0 (0.0–20.6)
Do you ever have an orgasm each time you would like?	Yes	135	63.7 (57.3–70.1)	10	7.4 (3.6–13.2)
	No	77	36.3 (29.9–42.7)	3	3.9 (0.8–10.9)
Do you ever have pain during intercourse?	Yes	10	4.7 (2.3–7.9)	0	0.0 (0.0–30.9)
	No	202	95.3 (92.1–97.7)	13	6.4 (3.5–10.8)
Do you ever have pain when you have achieved an erection?	Yes	5	2.4 (0.5–4.7)	1	20.0 (0.5–71.6)
	No	206	97.6 (95.3–99.5)	12	5.8 (3.1–9.9)
Do you ever have pain during ejaculation?	Yes	17	8.0 (4.7–12.0)	4	23.5 (6.8–49.9
	No	195	92.0 (88.0–95.3)	9	4.6 (2.1–8.6)

n: number of participants in each category

### 3.4 Semen quality and male genital schistosomiasis

Six of the 223 participants had abnormal sperm color: 5 (2.2%, 95% CI; 0.4%-4.5%) were brownish, and 1 (0.4%, 95% CI; 0.0%-1.3%) was cloudy. The mean sperm pH, volume, and count were 7.7 (95% CI; 7.7–7.8), 1.9 mL (95% CI; 1.6 ml-2.1 ml), and 48.0 million (95% CI; 44.2 million -50.0 million), respectively. Most of participants had normal semen pH (87.0%, 95% CI; 82.5%–91.5%) and volume (68.2%, 95% CI; 62.3%–74.4%). The majority of the 214 participants with sperm in their semen had normal sperm count (82.5%, 95% CI; 77.1%-87.4%), normal sperm motility (73.4%, 95% CI; 67.8%-79.2%), and normal sperm morphology (99.1%, 95% CI; 97.6%-100.0%). Approximately half of the participants had abnormal semen viscosity: 79 (35.4, 95% CI; 29.1%-42.2%) with hypoviscous semen and 20 (9.0%, 95% CI; 5.0%-12.7%) with hyperviscous semen (Table 6). Participants with brownish sperm had significantly higher prevalence of MGS infection (40%, 95% CI: 5.3%-85.3%).

**Table 6:** Results of characteristics of participants’ semen quality (N = 223) in Mtama district, Lindi from May to June 2022.

Variable	Category	n	% (95% CI)	Infected n, (%, 95% CI)	% of infected (95% CI)
Semen colour	Milky-white	217	97.3 (95.1–99.1)	11	5.1 (2.3–8.9)
	Brownish	5	2.2 (0.4–4.5)	2	40.0 (5.3–85.3)
	Cloudy	1	0.4 (0.0–1.3)	0	0.0 (0.0–97.5)
Semen pH	4.0–7.1	20	9.0 (5.4–13.0)	0	0.0 (0.0–16.8)
	7.2–8.0	194	87.0 (82.5–91.5)	13	6.7 (3.4 -.11.2)
	8.1–9.4	9	4.0 (1.8–6.7)	0	0.0 (0.0–33.6)
Semen volume (ml)	0.2–1.4	67	30.0 (24.2–36.4)	5	7.5 (2.5–16.6)
	1.5–5.0	152	68.2 (62.3–74.4)	8	5.3 (2.3–10.1)
	5.1–8.0	4	1.8 (0.4–3.6)	0	0.0 (0.0–60.2)
Semen viscosity	Hypoviscous	79	35.4 (29.1–42.2)	3	5.3 (0.8–10.7)
	Normal	124	55.6 (48.9–62.3)	10	8.1 (3.9–14.3)
	Hyperviscous	20	9.0 (5.0–12.7)	0	0.0 (0.0–16.8)
Sperm count (million)	0	9	4.0 (1.8–6.7)	0	0.0 (0.0–33.6)
	0.1–14.9	30	13.5 (59.4–17.9)	1	3.3 (0.08–17.2)
	15.0–120.	184	82.5 (77.1–87.4)	12	6.5 (3.4–11.1)
Sperm motility	Low	57	26.6 (20.8–32.7)	3	5.3 (1.1–14.6)
	Normal	157	73.4 (67.8–79.2)	10	6.4 (3.1–11.4)
Sperm morphology	Abnormal	2	0.9 (0.0–2.4)	0	0.0 (0.0–84.2)
	Normal	212	99.1 (97.6–100.0)	13	6.1 (3.3–11.3)

n: number of participants in each category

## 4 Discussion

Several urogenital infections can induce reproductive and sexual health problems in males by damaging testicular tissue and/or the genital ductal system [3]. These infections include sexually transmitted infections [3, 13]. In addition, some parasitic infections such as filariasis and schistosomiasis are suggested to cause male reproductive and sexual health problems [13]. Despite high schistosomiasis prevalence in many developing countries, MGS has received little attention as well as its contribution in causing male reproductive and sexual health problems [3, 13]. The current study attempted to determine the burden of MGS and the potential reproductive and sexual health problems that it could cause.

This study found that MGS is prevalent among men of reproductive health in southern Tanzania. The prevalence is lower than what has been reported along the southern shoreline of Lake Malawi (10.4%) [6], and in northern Madagascar (53%) [12]. Difference in sensitivities of the methods for detecting eggs in the sperm could explain the low prevalence observed in this study. While our study employed wet preparation method, the Madagascar study used filtration, whereas the Malawi study used multiple techniques and examined the entire collected specimen [6, 12]. Despite not having been extensively studied, the presence of *S*. *haematobium* eggs in semen only in 3.6% of all participants indicates that the prevalence of MGS is quite significant, similar to that of UGS in endemic areas [11].

The rates of MGS infection were found to be significantly higher among young adults (18–34 years old), followed by middle-aged adults (35–49 years). This pattern is consistent with the schistosomiasis intensity profile observed in other forms of schistosomiasis [24]. The profile revealed that schistosomiasis infection increases with age until it reaches a peak in the adolescent years, after which it begins to decline [24]. Apart from the development of immunity at older ages (due to the long period of exposure), another factor that has been proposed is the rate of water contact, which has been shown to increase with age until it reaches a peak at the age group of 5–9 years and then gradually decreases as age increases [24]. Schistosomiasis has been considered an occupational disease, particularly among adult male, with occupational exposures such as farming (peasants) and fishing [9]. However, in some cases, the risk of schistosomiasis infection may be higher in other occupational groups when other factors outweigh occupational exposure. For example, in this study, we discovered that petty businessmen have significantly higher MGS prevalence than peasants. This could be because, despite having access to tap water, in Mtama district many people only use tap water for drinking and cooking, preferring to use other water sources, primarily rivers and streams, for other activities such as bathing and washing to save money on their water bill [19]. The MGS prevalence was also found to be significantly higher among participants with no formal education. Similar finding was reported in other form of schistosomiasis in other at-risk population. A study to investigate the prevalence of UGS among women of reproductive age reported a high prevalence among those without formal education. Also, the study found that women without formal education had low awareness and knowledge of schistosomiasis transmission, prevention, and control [25].

According to the findings of this study, the prevalence of MGS is higher in males who stated that they have never caused pregnancy and have no children. Despite the fact that this study lacks the power to classify these groups of participants (those who have never caused pregnancy and those who do not have children) as infertile and to establish a cause-effect relationship between MGS and male infertility, the higher prevalence of MGS in these groups compared to their opposite groups should be taken seriously. MGS has been reported to be among of the cause of male infertility. It is evidenced that MGS is associated with seminal apoptosis and reduced semen quality [12], can cause intense epididymitis that can create obstructive azoospermia, inhibit spermatogenesis and cause irreversible male infertility with normal levels of gonadotrophins and testosterone [26], is associated with weak erection, rapid ejaculation and diminished libido [27]; and is associated with spermatogenesis arrest, which is a known cause of schistosomal inflammatory obstruction [28]. Therefore, contribution of MGS in causing male infertility should not be ignored.

Early symptoms of MGS include painful ejaculation and haemospermia [5]. In this study, males reported to experience pain during ejaculation had a high prevalence of male MGS. Despite the fact that there was no visible blood in any of the participants’ semen, we did find brown-colored semen in five of them, two of whom had MGS. The brownish colour of the semen is sometimes thought to be an indicator of blood in the semen [21].

## 5 Conclusion

MGS, like UGS, is prevalent condition in southern Tanzania. Despite the fact that our study design lack the power to establish cause and effect relationship between MGS and reproductive and sexual health issues, the study discovered that MGS is present among participants who stated or were observed to have some reproductive and sexual health issues, such as infertility, painful ejaculation, and brownish sperm. This highlights the need for more research to investigate the association of MGS and male reproductive and sexual health for the improvement of health services among males.

### 5.1 Study limitations

One limitation of this study is that it may have underestimated the prevalence of MGS because of the low sensitivity of the technique employed to detect *Schistosoma* eggs in the semen samples. Furthermore, the study was unable to rule out other possible causes of blood in semen.

## Supporting information

S1 Checklist STROBE statement—checklist of items that should be included in reports of observational studies. (DOCX)

## References

[1] World Health Organization. Ending the neglect to attain the Sustainable Development Goals–a road map for neglected tropical diseases 2021–2030. Geneva; 2020.

[2] Aula OP, McManus DP, Jones MK, Gordon CA. Schistosomiasis with a focus on Africa. Trop Med Infect Dis. 2021;6(3):1–40. doi: 10.3390/tropicalmed6030109 34206495 PMC8293433

[3] Abdel-Naser BM, Altenburg A, Zouboulis CC, Wollina U. Schistosomiasis (bilharziasis) and male infertility. Andrologia. 2018;e13165. doi: 10.1111/and.13165 30276841

[4] Ross A, Chau T, Inobaya M, Olveda R, Li Y, Harn D. A new global strategy for the elimination of schistosomiasis. Int J Infect Dis. 2017;54:130–7. doi: 10.1016/j.ijid.2016.09.023 27939558

[5] Mazigo HD, Uisso C, Kazyoba P, Nshala A, Mwingira UJ. Prevalence, infection intensity and geographical distribution of schistosomiasis among pre-school and school aged children in villages surrounding Lake Nyasa, Tanzania. Sci Rep. 2021;11(1):1–11. 33432079 10.1038/s41598-020-80317-x PMC7801377

[6] Kayuni SA, Alharbi MH, Makaula P, Lampiao F. Male genital schistosomiasis along the shoreline of Lake Malawi: baseline prevalence and associated knowledge, attitudes and practices mmong local fishermen in Mangochi. Front Public Heal. 2021;9(590695). 10.3389/fpubh.2021.590695 PMC8175656 34095041

[7] Phillips AE, Gazzinelli-Guimarães PH, Aurelio HO, Dhanani N, Ferro J, Nala R, et al. Urogenital schistosomiasis in Cabo Delgado, northern Mozambique: Baseline findings from the SCORE study. Parasites and Vectors. 2018;11(1):1–10. 29316983 10.1186/s13071-017-2592-8 PMC5761122

[8] Hong ST. Review of recent prevalence of urogenital schistosomiasis in Sub-Saharan Africa and diagnostic challenges in the field setting. Life. 2023;13(8). doi: 10.3390/life13081670 37629527 PMC10456001

[9] Gyapong M, Theobald S. The sexual and reproductive health issue you’ve probably never heard of…. Why is one of the most common gynaecological conditions in sub-Saharan Africa, schistosomiasis, misunderstood, under-researched and under-reported? [Internet]. Open Democracy. 2015 [cited 2021 Oct 25]. Available from: https://www.opendemocracy.net/en/5050/

[10] Madden F. Two rare manifestations of bilharziosis. Lancet. 1911;178(4593):754–5.

[11] Kayuni S, Lampiao F, Makaula P, Juziwelo L, Lacourse EJ, Reinhard-rupp J, et al. A systematic review with epidemiological update of male genital schistosomiasis (MGS): A call for integrated case management across the health system in sub-Saharan Africa. Parasite Epidemiol Control. 2019;4:e00077. doi: 10.1016/j.parepi.2018.e00077 30662962 PMC6324017

[12] Leutscher PDC, Høst E, Reimert CM. Semen quality in Schistosoma haematobium infected men in Madagascar. Acta Trop. 2009;109(1):41–4. doi: 10.1016/j.actatropica.2008.09.010 18950598

[13] Stecher CW, Kallestrup P, Kjetland EF, Vennervald B, Petersen E. Considering treatment of male genital schistosomiasis as a tool for future HIV prevention: a systematic review. International Journal of Public Health. 2015; 60: 839–48. doi: 10.1007/s00038-015-0714-7 26298443

[14] Ribeiro AR, Luis C, Fernandes R, Botelho MC. Schistosomiasis and Infertility: What Do We Know?. Trends in Parasitology. 2019; 35: 964–71. doi: 10.1016/j.pt.2019.09.001 31623951

[15] Adisa J, Egbujo EM, Yahaya BA, Echejoh G. Primary infertility associated with schitosoma mansoni: A case report from the Jos Plateau, north central Nigeria. Afr Health Sci. 2012;12(4):563–5. doi: 10.4314/ahs.v12i4.26 23515635 PMC3598301

[16] Gouvras A. Urogenital schistosomiasis and the impact on sexual and reproductive health [Internet]. BMC BugBitten. 2018 [cited 2021 Oct 19]. Available from: https://blogs.biomedcentral.com/bugbitten/2018/07/13/

[17] Larsen U, Masenga G, Mlay J. Infertility in a community and clinic-based sample of couples in Moshi, northern Tanzania. East African Medical Journal. 2006; 83(1): 10–17. doi: 10.4314/eamj.v83i1.9355 16642745

[18] Mushi V, Zacharia A, Shao M, Mubi M, Tarimo D. Persistence of Schistosoma haematobium transmission among school children and its implication for the control of urogenital. PLoS One. 2022;17(2): e0263929. 35167622 10.1371/journal.pone.0263929 PMC8846507

[19] Mushi V, Zacharia A, Shao M, Mubi M, Tarimo D. Prevalence and risk factors of urogenital schistosomiasis among under-fives in Mtama District in the Lindi region of Tanzania. PLoS Negl Trop Dis. 2022;16(4):e0010381. doi: 10.1371/journal.pntd.0010381 35442997 PMC9060350

[20] Daniel W. Biostatics a foundation for analysis in the health science. 6th ed. New York: John Willey and Sons Inc; 1995

[21] Leutscher P, Ramarokoto CE, Reimert C, Feldmeier H, Esterre P, Vennervald BJ. Community-based study of genital schistosomiasis in men from Madagascar. Lancet. 2000;355(9198):117–8. doi: 10.1016/S0140-6736(99)04856-4 10675174

[22] Björndahl L, Mortimer D, Barratt CLR, Castilla JA, Menkveld R, Kvist U, et al. Basic semen analysis. In: A Practical Guide to Basic Laboratory Andrology. Cambridge University Press; 2010; 33–76.

[23] WHO. Bench aids for diagnosis of intestinal parasites. 2nd ed. Geneva: World Health Organization (2019). p. 32.

[24] Kura K, Hardwick RJ, Truscott JE, Anderson RM. What is the impact of acquired immunity on the transmission of schistosomiasis and the efficacy of current and planned mass drug administration programmes? PLoS Negl Trop Dis. 2021;15(12):1–17. doi: 10.1371/journal.pntd.0009946 34851952 PMC8635407

[25] Ngassa N, Zacharia A, Lupenza ET, Mushi V, Ngasala B. Urogenital schistosomiasis: prevalence, knowledge and practices among women of reproductive age in Northern Tanzania. IJID Reg. 2023;6:15–23. doi: 10.1016/j.ijregi.2022.09.013 36578524 PMC9791118

[26] Kini S, Dayoub N, Raja A, Pickering S, Thong J. Schistosomiasis-induced male infertility. BMJ Case Rep. 2009; 2009: bcr0120091481. doi: 10.1136/bcr.01.2009.1481 21857876 PMC3027935

[27] Ghoneim MA. Bilharziasis of the genitourinary tract. BJU Int Suppl. 2002;89(1):22–30. doi: 10.1046/j.1464-4096.2001.138.138.x 11876729

[28] Omer EFE. Inflammatory conditions and semen quality among subfertile Sudanese males. Trop Doct. 1985;15(1):27–8. doi: 10.1177/004947558501500114 3976003

